# Elevated MST1 leads to apoptosis via depletion of YAP1 in cardiomyocytes exposed to high glucose

**DOI:** 10.1186/s10020-021-00267-6

**Published:** 2021-02-10

**Authors:** Dongmei Su, Yanhua Li, Lina Guan, Qian Li, Cuige Shi, Xu Ma, Yonghui Song

**Affiliations:** 1 Department of Genetics, Center for Genetics, National Research Institute for Family Planning, Health Department, 12, Dahuisi Road, Haidian, Beijing, 100081 China; 2 Graduate School, Peking Union Medical College, Beijing, China; 3 Department of Teaching and Research of Obstetrics and Gynecology, Shandong Medical College, Linyi, Shandong China; 4 Department of Obstetrics, Linyi People’s Hospital, 27, Jiefang Road, Linyi, 276003 Shandong China

**Keywords:** High glucose, Cardiomyocyte apoptosis, MST1, YAP1, Survivin

## Abstract

**Background:**

Gestational diabetes mellitus is a risk factor for congenital heart defects. The article aimed to investigate the expression and roles of MST1, YAP1, Last1/2 and Survivin in modulating HG-induced cardiomyocyte apoptosis and maternal diabetes-induced heart abnormality.

**Methods:**

Diabetes mellitus was induced in rats using streptozotocin. The protein expression and phosphorylation analysis in fetal heart tissue was assessed by western blot and immunohistochemical staining. Hoechst 33342 staining assay was performed to explore H9C2 apoptosis. The gene and protein expression in H9C2 cells was assessed by quantitative PCR and western blot. Knockdown of gene expression was assessed by RNA interference.

**Results:**

Our results revealed that increased MST1 protein levels in the heart tissues of the offspring of diabetic rats in vivo and in H9C2 cardiomyocytes under HG treatment in vitro, respectively. Knockdown and overexpression experiments showed that MST1 played a key role in mediating HG-induced apoptosis in cardiomyocytes. Downregulation of YAP1 was associated with HG-induced, MST1-mediated cardiomyocyte apoptosis. Further study showed that MST1 downregulated the protein level of YAP1 through mediation of YAP1 phosphorylation on Ser127 and Ser397; this process also required LATS1/2 participation. MST1 overexpression increased the phosphorylation levels of LATS1/2, which were also shown to be increased in the heart tissues of diabetic offspring. We also found that YAP1 mediated the expression of Survivin during HG-induced apoptosis, and the Survivin-inhibitor YM155 partially inhibited the role of YAP1 in suppressing apoptosis induced by HG in cardiomyocytes.

**Conclusion:**

These findings reveal a regulatory mechanism of MST1/YAP1/Survivin signaling in modulating cardiomyocyte apoptosis in vitro and maternal diabetes-induced congenital heart defects in vivo.

## Introduction

Congenital heart disease (CHD) is a common defect that clinically manifests as anomalies in the heart and great vessels (Miller et al. 2016). Postnatal studies have revealed that the mothers of many infants with abnormalities involving CHD have diabetes (Priest et al. 2015; Correa et al. 2008). Indeed, increasing evidence shows that exposure to hyperglycemia in utero induces not only short-term consequences, but also long-term effects such as congenital birth defects and metabolic syndrome (Metzger et al. 2008; Simeoni and Barker 2009). These defects are most common and severe in the central nervous and cardiovascular systems (Agoudemos et al. 2011; Zhao 2010). The molecular basis of CHD pathogenesis in pregestational diabetes remains largely uncharacterized. Previous studies described diabetes-induced congenital malformations that occur during heart development, with some reporting increased numbers of apoptotic myocardial cells that participate in gestational diabetes mellitus-induced heart abnormalities (Moazzen et al. 2014; Bohuslavova et al. 2013; Gutierrez et al. 2009). However, the molecular mechanisms and factors responsible for the high incidence of CHD in pregestational diabetes require further elucidation.

The Hippo signaling pathway was originally discovered through a series of genetic mosaic screens for genes augmenting cell proliferation and organ size in Drosophila (Harvey et al. 2003). Hippo pathway core components (MST1/2 and YAP) are important for development and tissue homeostasis, while aberrant signaling through the Hippo pathway has been implicated in multiple pathologies, including cancer (Lee and Machner 2018). In canonical Hippo signaling, as upstream activator, Mammalian sterile 20-like kinase 1/2 (MST1/2) kinases associate with Salvador family WW domain containing protein 1 (SAV1) and Mps one binder kinase activator-like 1A and 1B (MOB1A/B or collectively, MOB1) to phosphorylate large tumour suppressor 1/2 (LATS1/2). LATS1/2 subsequently phosphorylate Yes-associated protein (YAP) to lead to its cytoplasmic retention and degradation, and subsequent transcription suppression of its target genes (Taha et al. 2018; Yuan et al. 2017). In non-canonical signaling, MST1 mediates AMPK phosphoryaltion, then AMPK directly phosphorylates YAP1 at Ser94 (Feng et al. 2018; Hong et al. 2016). Whether the canonical or non-canonical signaling mediated YAP1 phosphorylation in cardiomyocytes under high glucose (HG) treatment is investigated in this study.

MST1, a key upstream regulator of Hippo pathway activation, plays an important role in regulating cell growth, proliferation and apoptosis (Bitra et al. 2017). Its C terminus shares only 60% amino acid identity with MST2. Many studies showed that MST1 were related to the heart disease. MST1 regulates heart size by activating downstream target kinases, Lats1/2, thereby inhibiting compensatory cardiomyocyte growth (Matsui et al. 2017). However, little is known about the expression profile of MST1 or its role in the pathogenesis of maternal diabetes-induced fetal heart abnormalities and cardiomyocyte apoptosis.

YAP1, as a transcriptional modulator, mediates cell proliferation and apoptosis through many target genes, such as CyclinD1 (Wong et al. 2016) and Survivin (Rosenbluh et al. 2012), in response to stressors in different cell types. Cyclin D1 functions as a checkpoint at G1/S phase transition, decreased percentage of Cyclin D1 expression is associated with cancer cells apoptosis (Yudhani et al. 2019). Survivin, a representative member of the inhibitor of apoptosis protein (IAP) family is highly expressed in many tumor types (Luo et al. 2019). Abnormal expression level of Survivin in blood has been studied as a potential biomarker in several tumors (Samarkos et al. 2018). YAP1 interacts with both β-catenin and TBX5 to regulate Survivin expression in cancer cell lines (Rosenbluh et al. 2012). Although Survivin and CyclinD1 have been well studied in tumors, their roles in heart disease are poorly understood. The objective of this study is to examine whether YAP1 regulates cardiomyocyte apoptosis by regulating Survivin or CyclinD1 in cardiomyocyte apoptosis and abnormal heart development under HG.

The present study investigated molecular mechanisms underlying cardiomyocyte apoptosis and maternal diabetes-induced CHD in vivo and in vitro. By analyzing the expression pattern of MST1, YAP1 and Survivin after exposure to HG, we aimed to identify the molecular mechanism of these proteins and the Hippo pathway in modulating HG-induced cardiomyocyte apoptosis and maternal diabetes-induced CHD.

## Materials and methods

### Cell culture, plasmids and transfection

H9C2 rat cardiomyoblast cells were maintained in Dulbecco's modified Eagle's medium supplemented with 10% fetal bovine serum, cardiac myocyte growth supplement, 100 mg/mL penicillin, and 100 mg/mL streptomycin in a humidified atmosphere containing 5% CO_2_ at 37 °C.

MST1 and YAP1 overexpression plasmids were purchased from Polepolar Research Company (China). Transient transfection was performed using Lipofectamine 3000 (Invitrogen) procedures.

### Hoechst 33342 stain apoptosis assay

Apoptosis was assessed through observation of morphologic changes in cell nuclei stained with Hoechst 33342 (Sigma) and examined under fluorescence microscopy. In 5 randomly selected fields, the numbers of apoptotic nuclei were counted.

### RNA interference (RNAi)

The small interfering RNA (siRNA) sequences were 5′-GCCGAGCCTTCCACTACAATA-3′ for MST1-targeting (Wu et al. 2016) and 5′-CGTCAACATGGCTTTCACC-3′ for a negative control. Oligonucleotides yielding small hairpin RNAs targeting MST1 and negative control sequences were synthesized and cloned into the pSilencer 4.1-CMV Neo vector (Ambion, USA) between BamHI and HindIII sites according to the manufacturer’s instructions. The constructed siRNA plasmids were transfected using Lipofectamine 3000 (Invitrogen) procedures.

### RNA isolation, reverse transcription and real-time PCR

RNA was extracted from the heart tissues using TRIzol reagent (Invitrogen) according to the manufacturer's protocol. Complementary DNA was synthesized from 2 μg RNA using an RNA PCR kit (TaKaRa, Dalian, China). Quantitative real-time PCR (qRT-PCR) was performed on an ABI Prism7500 Sequence Detection System (Applied Biosystems), following the manufacturer's protocol and using SYBR Green (TaKaRa, Osaka, Japan) as a double-stranded DNA-specific fluorescent dye. The primer pairs for YAP1 were: sense, 5′-TCGGCAGGCAATACGGAATA-3′; and antisense, 5′-CATGCTGAGGCCACTGTCTGT-3′ (Xie et al. 2012). The primer pairs for MST1 were: sense, 5′-CATGGCTCAGGTGAACAGTAT-3′; and antisense, 5′-GGTCTCTGGGTCCAAAGTATAAC-3′. The ß-actin primer pairs were: sense, 5′-TCGTGCGTGACATTAAGGAG-3′; and antisense, 5′-ATGCCAGGGTACATGGTGGT-3′.

### Animal modeling and isolation of embryo hearts

Diabetes mellitus was induced in rats using streptozotocin as described in our previous study (Su et al. 2016). At embryonic stage E15.5, pregnant rats were euthanized and the diabetes-exposed fetuses were collected by caesarean section for examination of the hearts. The experimental protocol was in compliance with the National Institutes of Health Guide for Care and Use of Laboratory Animals.

### Western blotting

H9C2 cells and frozen fetal heart tissues were lysed in buffer. Total protein (about 40 µg) was applied to a 12% sodium dodecyl sulphate-polyacrylamide electrophoresis gel. After electrophoresis, the polyvinylidene fluoride membrane was incubated with the following antibodies: anti-MST1 (Abcam), anti-YAP1 (Abcam), anti-YAP(Ser397) (Cell Signaling), anti-YAP(Ser127) (Cell Signaling), anti-LATS1/2 (MyBioSource), anti-LATS1/2-(Thr1079/1041) (Biorbyt), anti-Survivin (Abcam) and anti-β-actin (Sigma Aldrich). Signals were visualized using a chemiluminescent substrate method with a SuperSignal West Pico Kit (Pierce Biotechnology, USA). The experiments were repeated 3 times. We have quantified the results from 3 independent western blots by Image J software.

### Immunohistochemistry (IHC) staining

Paraffin sections were deparaffinized and hydrated using a xylene and graded alcohol series. After rinsing with water, the sections were boiled for 10 min in 0.1 M citric acid (pH 6.1) and allowed to cool to room temperature. Sections were washed with phosphate-buffered saline, placed in 0.3% H_2_O_2_ to quench endogenous peroxidase activity, and washed again. The sections were incubated with normal blocking serum for 1 h, and then with anti-MST1 and anti-YAP1 antibodies (both from Abcam) overnight. After washing, the sections were incubated for 1 h with a biotinylated secondary antibody followed by incubation with a preformed complex of avidin and biotinylated peroxidase. Finally, the sections were incubated in a peroxidase substrate solution (diaminobenzidine tetrahydrochloride) until the desired stain intensity developed, rinsed with water, cleared and mounted.

### Statistical analysis

Student’s t-test and analysis of variance were used to calculate statistical significance. A p value < 0.05 was considered to indicate significance. Significance levels were set as *p < 0.05; ^#^p < 0.05; **p < 0.01; ^##^p < 0.01. Error bars denote standard deviation.

## Results

### Upregulation of MST1 in cardiomyocytes of diabetic offspring in vivo and in vitro

**Fig. 1:**
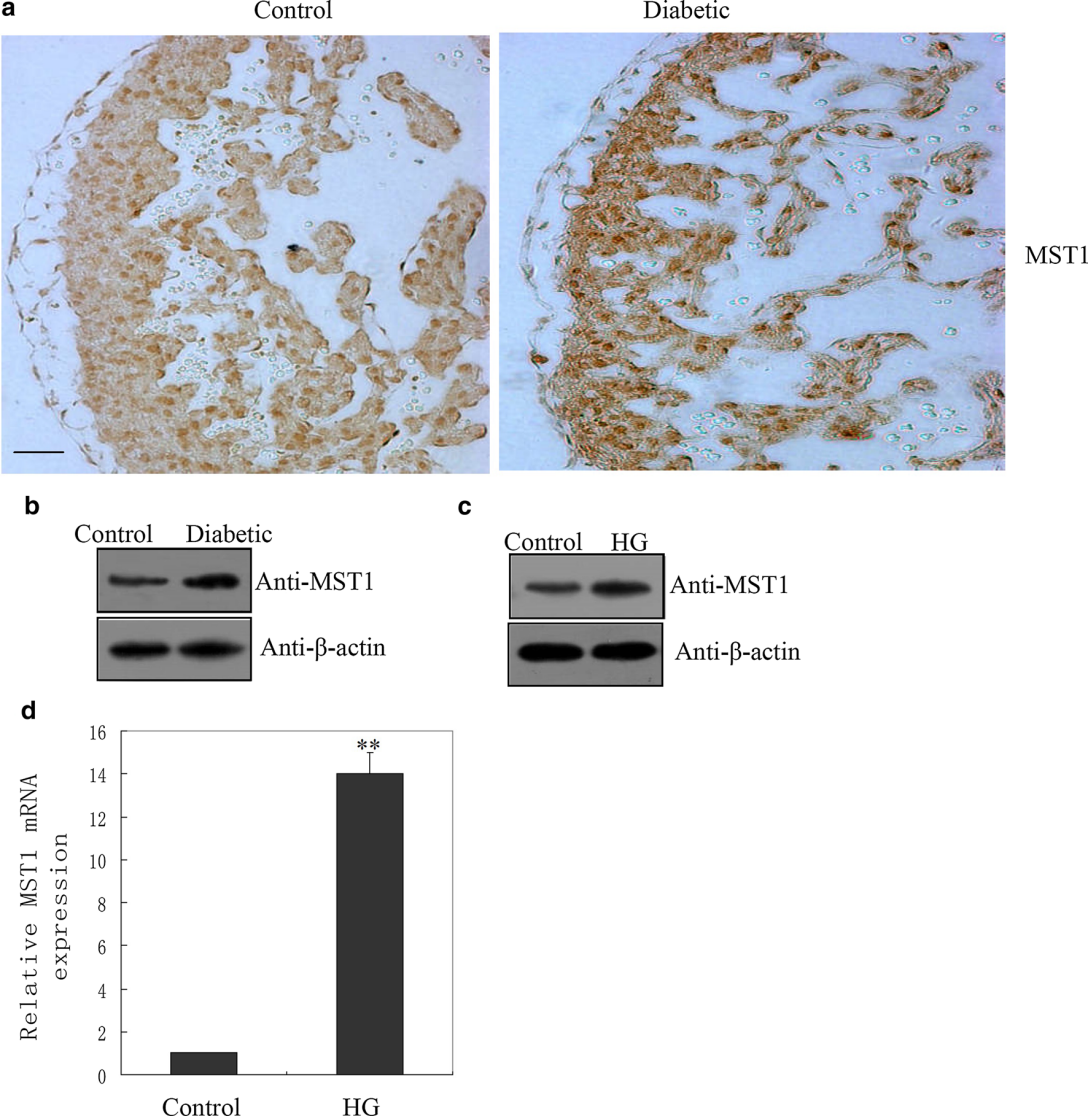
Increase in mammalian sterile 20-like kinase 1 (MST1) in the cardiomyocytes of diabetic offspring in vivo. **a** Immunostaining of MST1 in the myocardium of normal and diabetes-exposed embryos at E15.5 (n = 3). Scale bar: 50 µm. **b** Western blotting with the indicated antibodies in the diabetes and non-diabetes groups (n = 8 each) confirmed the increase in MST1 protein in heart tissues. β-Actin was used as an internal reference control. Western blotting of MST protein (**c**) and real-time PCR of MST1 mRNA expression (**d**) in cardiomyocytes after 2 days of high glucose (HG) treatment. Data are based on three independent experiments

IHC revealed an increase in MST1 protein level in the heart tissues of diabetes-exposed embryos (Fig. 1a), that was confirmed by western blotting (Fig. 1b and Additional file 1: Fig. S1A). A previous study indicated a marked increase in apoptotic cardiomyocytes in response to HG exposure in vitro (Su et al. 2016). In this study, MST1 protein level was increased in H9C2 cardiomyocytes after exposure to HG (Fig. 1c and Additional file 1: Fig. S1B). In addition, MST1 mRNA level was increased in H9C2 cardiomyocytes after exposure to HG, as shown in Fig. 1d.

### MST1 played a key role in mediating HG-induced cardiomyocyte apoptosis

**Fig. 2:**
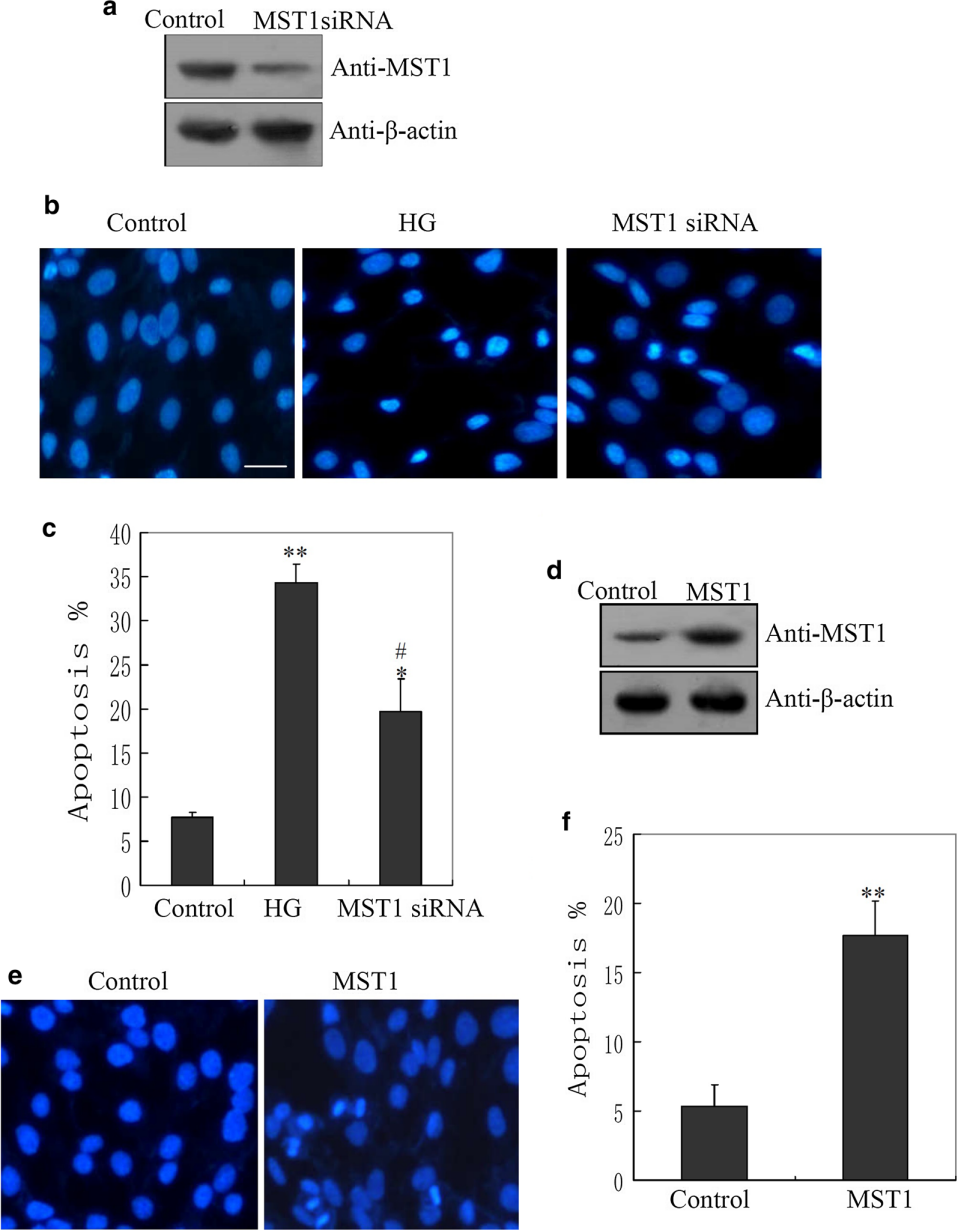
Effects of mammalian sterile 20-like kinase 1 (MST1) on high glucose (HG)-induced cardiomyocyte apoptosis. **a** Western blotting confirmation of MST1 knockdown by an MST1-specific or control small interfering RNA. **b** Hoechst 33342 staining assay showing restrained HG-induced cell apoptosis under knockdown of MST1. **c** Apoptosis rates were calculated based on ≥ 100 cells and analyzed after HG treatment; *p < 0.05 and **p < 0.01 versus the untreated group; ^#^p < 0.05 and ^##^p < 0.01 versus the HG-treated group. **d** Western blotting confirmation of ectopic overexpression of MST1 after transfection of H9C2 cells. **e** Hoechst 33342 stain apoptosis assay showing the effect of MST1 overexpression on apoptosis in H9C2 cells; (F) Apoptosis rates were calculated based on ≥ 100 cells and analyzed after MST1 transfection; *p < 0.05 and **p < 0.01 versus the non-transfected group

The effect of MST1 on cardiomyocyte apoptosis was investigated using an RNAi approach. Western blotting showed that endogenous MST1 protein expression was markedly reduced by transfection of H9C2 cells with a MST1-specific siRNA plasmid (Fig. 2a and Additional file 2: Fig. S2A). Knockdown of endogenous MST1 also reduced HG-induced apoptosis of cardiomyocytes (Fig. 2b, c). We then constructed an MST1 overexpression plasmid and used western blotting to confirm that transfected cells had a marked increase in MST1 protein expression (Fig. 2d and Additional file 2: Fig. S2B). Overexpression of MST1 increased the ratio of apoptotic cells (Fig. 2e, f).

### The decreased protein levels of of YAP1 was associated with HG-induced apoptosis in cardiomyocytes in vivo and in vitro

**Fig. 3:**
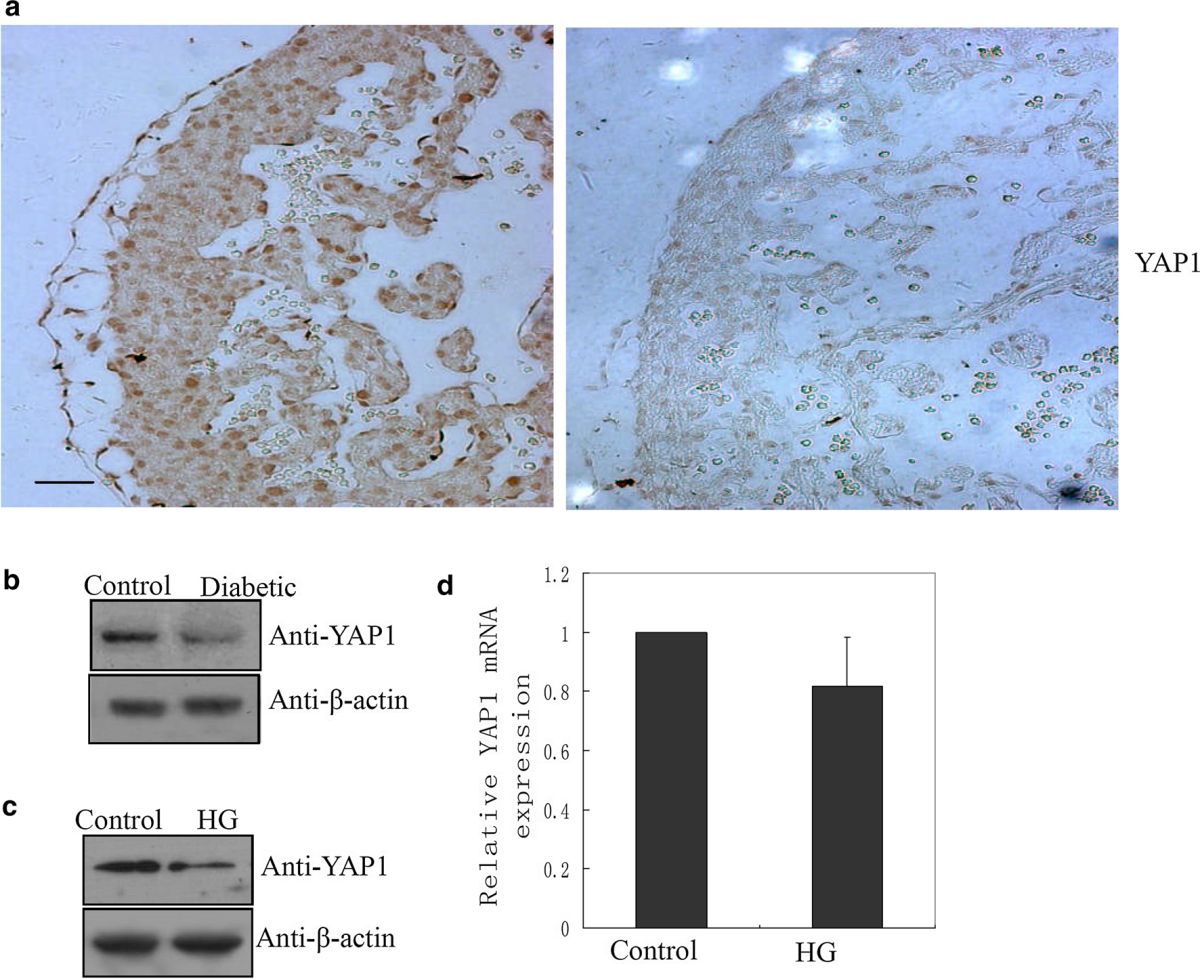
Association between knockdown of yes-associated protein 1 (YAP1) and mammalian sterile 20-like kinase 1 (MST1)-mediated cardiomyocyte apoptosis. **a** Immunohistochemical staining showed of YAP1 in the myocardium of normal and diabetes-exposed embryos (n = 3). Scale bar: 50 µm. **b** Western blotting with the indicated antibodies confirming the decrease in YAP1 protein in fetal hearts at E15.5 from the normal and diabetic groups (n = 8 each). β-actin was used as an internal reference control. **c** Western blotting of YAP1 protein in cardiomyocytes after 2 days of high glucose (HG) treatment; experiments were replicated 3 times. **d** The mRNA expression of YAP1 in cardiomyocytes after HG treatment; data are based on three independent experiments

Then, we studied the expression of YAP1, the core protein of Hippo pathway fetal heart tissue. Immunohistochemistry showed that the protein level of YAP1 were decreased in the heart tissue of diabetic offspring (Fig. 3a). Western blotting confirmed the decreased YAP1 protein levels in the heart tissue of diabetic offspring (Fig. 3b and Additional file 3: Fig. S3A). In vitro, exposure to HG resulted in decreased YAP1 protein in cardiomyocytes (Fig. 3c and Additional file 3: Fig. S3B), but did not lead to an obvious change in YAP1 mRNA levels (Fig. 3d).

### Down-regulation of YAP1 participated in MST1-mediated apoptosis in cardiomyocytes

**Fig. 4:**
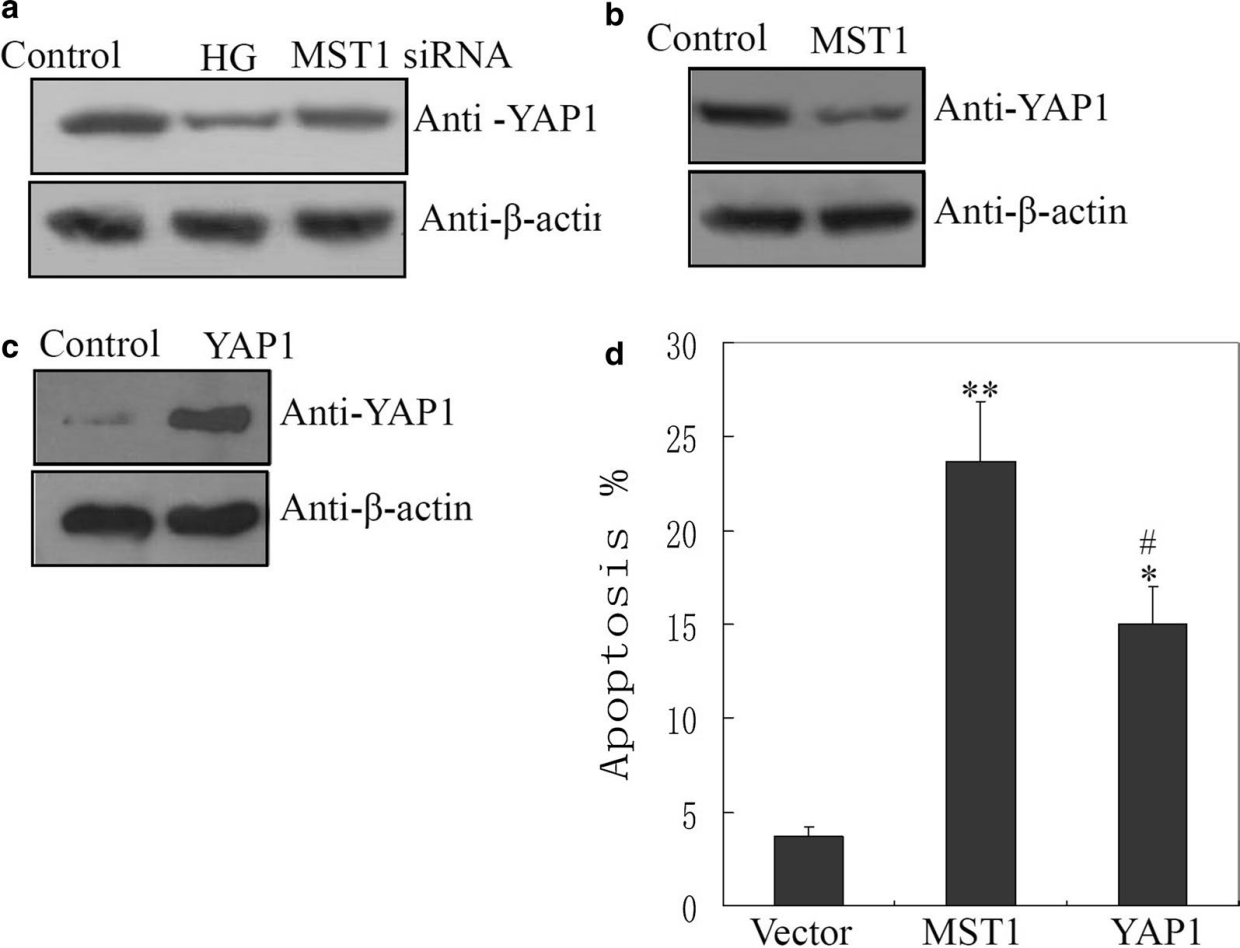
Down-expression of YAP1 are associated with MST1-mediated cardiomyocyte apoptosis. **a** The decrease YAP1 protein level in response to HG was remarkably suppressed when H9C2 cells were transfected with MST1 siRNA for western blotting. **b** Western blotting estimated of the protein level of YAP1 after transfection with MST1 expression plasmid. **c** Western blotting confirmation of the ectopic expression of YAP1 in H9C2 cells after transfection with YAP1 expression plasmid. **d** Over-expression of YAP1 suppressed the increase apoptotic rate in H9C2 cells, which was mediated by MST1 induced by HG

We next determined whether MST1 mediated YAP1 protein expression in vitro. Western blotting revealed that down-regulated YAP1 protein level in response to HG treatment was rescued in cardiomyocytes after transfection with MST1 siRNA (Fig. 4a and Additional file 4: Fig. S4A). Moreover, MST1 over-expression down-regulated the protein level of YAP1 (Fig. 4b and Additional file 4: Fig. S4B). Next we asked whethert removal of YAP1 leads to apoptosis and that adding it inhibited MST1′s role in mediating apoptosis upon HG exposure to test this, we constructed a YAP1 overexpression plasmid. Western blotting results confirmed that the protein level of YAP1 increased obviously after transfection with YAP1 plasmid. (Fig. 4c and Additional file 4: Fig. S4C). Apoptosis assay showed that the ability of MST1 to induce apoptosis could be effectively inhibited by over-expression of YAP1 (Fig. 4d). These results indicated that down-regulation of YAP1 participated in MST1-mediated apoptosis in cardiomyocytes.

### MST1 mediated YAP1 phosphorylation through phosphorylation of Lats1/2

**Fig. 5:**
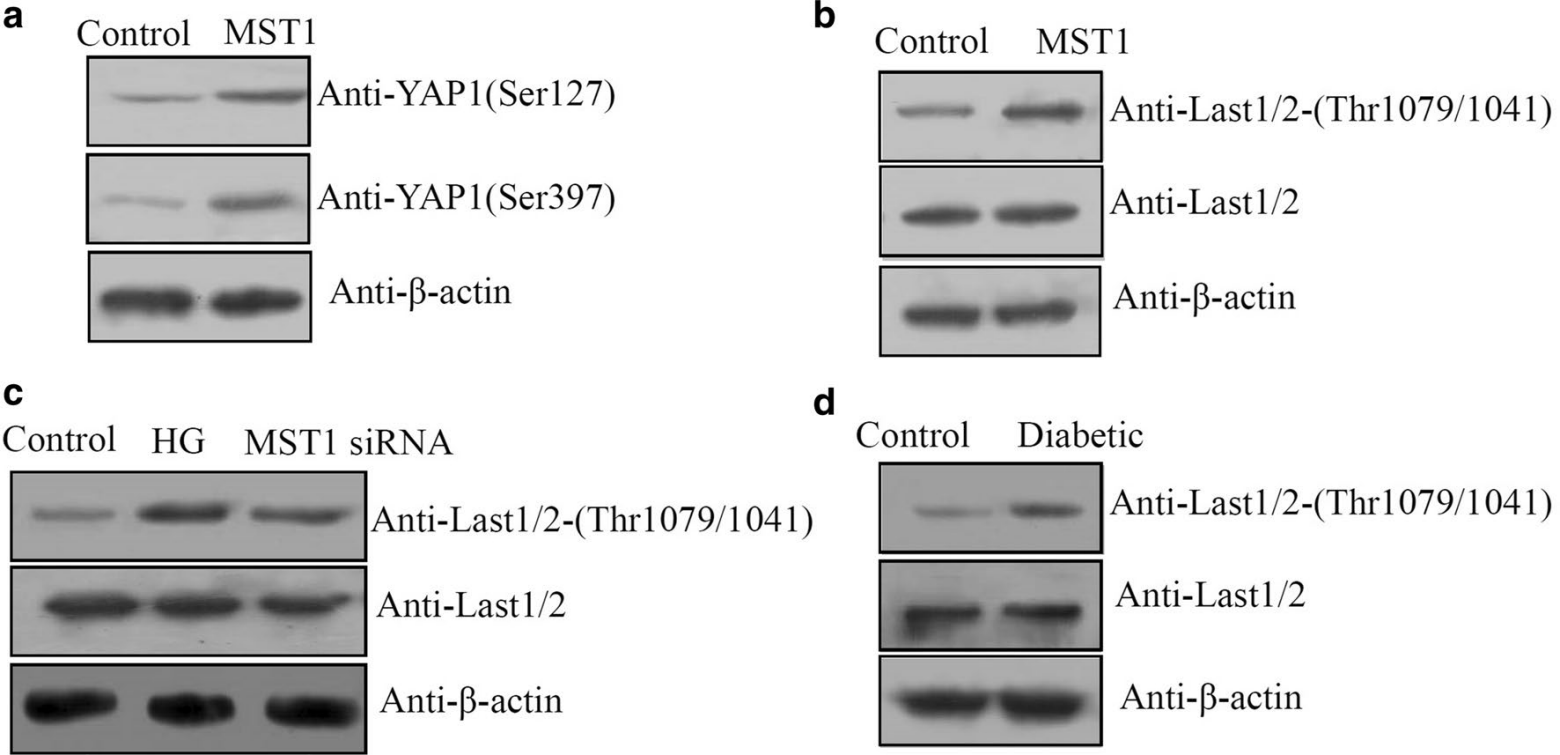
Mediation of yes-associated protein 1 (YAP1) phosphorylation by mammalian sterile 20-like kinase 1 (MST1) in response to high glucose (HG) in cardiomyocytes. Western blotting-estimates of: phosphorylation levels of **a** YAP1 and **b** large tumor suppressor kinase 1 and 2 (LATS1/2) at Thr1079 and 1041 in H9C2 cells after transfection with the MST1 overexpression plasmid. **c** Knockdown of MST1 suppressed the HG-induced increased phosphorylation of LATS1/2 at Thr1079 and 1041; **d** LATS1/2 protein and phosphorylation levels in fetal hearts from the diabetic and normal groups (n = 8 each)

To investigate how MST1 mediated YAP1 protein expression in vitro, western blotting result showed that YAP1 phosphorylation at both Ser127 and Ser397 was increased in cardiomyocytes transfected with the MST1 overexpression plasmid (Fig. 5a and Additional file 5: Fig. S5A). To better understand the relationship between MST1 and YAP1, we examined the phosphorylation of Lats1/2, direct upstream mediators of YAP1 phosphorylation, which phosphorylate YAP1 at Ser127 and Ser397 (Jang et al. 2017; Zhao et al. 2010; Varelas 2014). As shown in Fig. 5b, when MST1 was overexpressed, phosphorylation of Lats1/2 (phospho-Thr1079/1041) was increased in cardiomyocytes. Furthermore, the increase in phosphorylation level of Lats1/2 in response to HG treatment was suppressed in cardiomyocytes after transfection with MST1 siRNA (Fig. 5c and Additional file 5: Fig. S5B). Western blotting also indicated an increase in Lats1/2 phosphorylation levels in the heart tissues of diabetic offspring (Fig. 5d and Additional file 5: Fig. S5C). These results suggested that MST1 mediated the phosphorylation of YAP1 through Lats1/2.

### Survivin was targeted by YAP1 in response to HG in cardiomyocytes

**Fig. 6:**
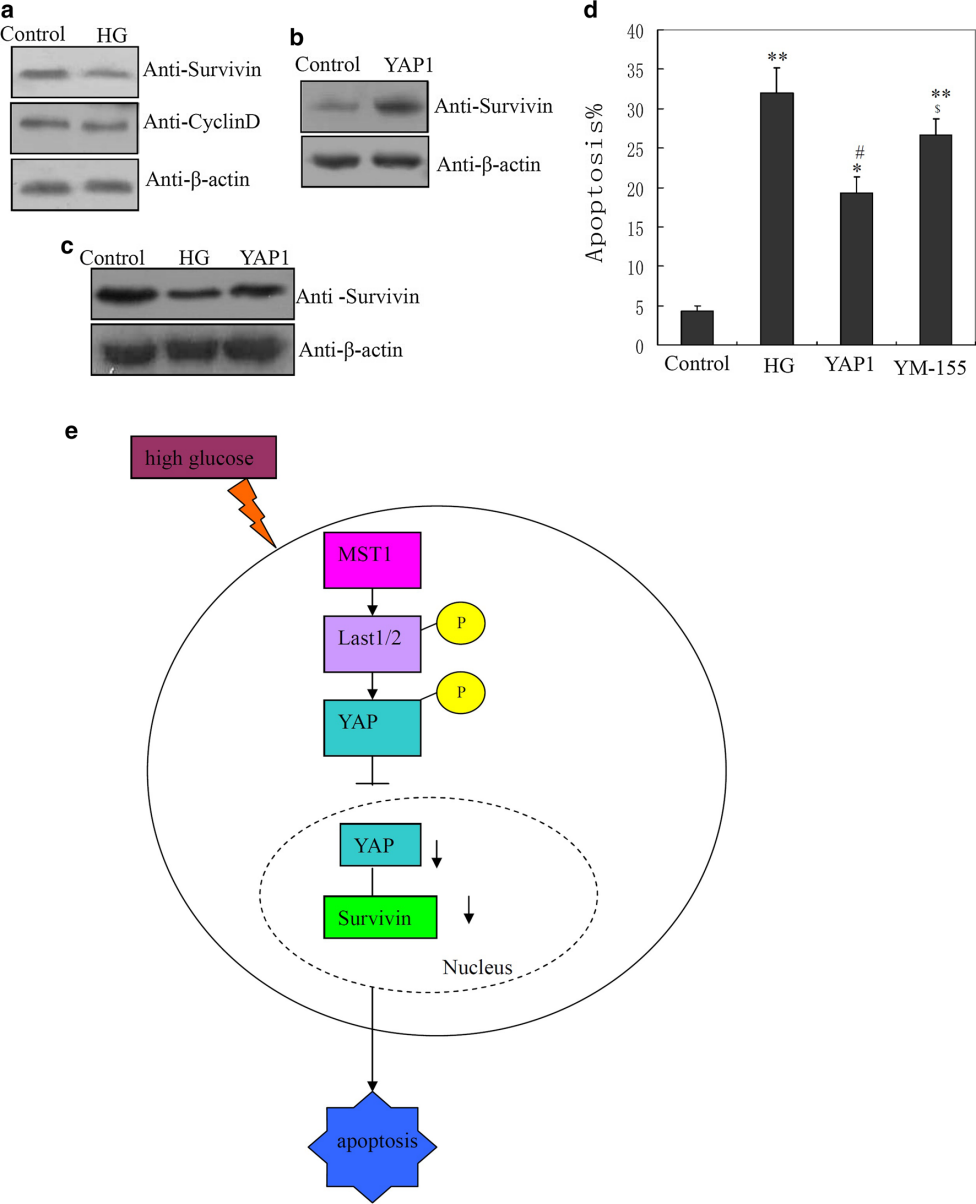
Survivin targeted by yes-associated protein 1 (YAP1) in response to high glucose (HG) in cardiomyocytes. Western blotting showing: **a** Survivin and CyclinD1 protein expression in cardiomyocytes after 2 days of HG treatment (experiments were replicated 3 times); effect of YAP1 overexpression on **b** upregulation of Survivin protein (n = 3) and **c** suppression of HG-mediated downregulation of Survivin (n = 3). **d** Hoechst 33,342 staining showing inhibitory effect of YM155 on the capacity of YAP1 to suppress HG-induced apoptosis; *P < 0.05 and **P < 0.01 compared with the untreated group; ^#^P < 0.05 and ^##^P < 0.01 compared with the HG-treated group (n = 3); ^$^P < 0.05 and ^$$^P < 0.01 compared with the YAP1 plasmid-transfected group. **e** Sketch diagram showing the regulatory mechanism of the mammalian sterile 20-like kinase 1 (MST1)/large tumor suppressor kinase 1 and 2 (LATS1/2)/YAP1/Survivin pathway in modulating cardiomyocyte apoptosis and maternal diabetes-induced heart defects

The protein levels of the YAP1 target genes CyclinD1 and Survivin in cardiomyocytes under HG treatment were detected by western blotting. As shown in Fig. 6a and Additional file 6: Fig. S6A, the protein level of Survivin was decreased in cardiomyocytes after exposure to HG, but there was little change in CyclinD1. Survivin was upregulated by YAP1 overexpression (Fig. 6b and Additional file 6: Fig. S6B), and the HG-mediated decrease in the protein level of Survivin was inhibited by overexpression of YAP1 (Fig. 6c and Additional file 6: Fig. S6C). Furthermore, treatment with YM155, an effective inhibitor of Survivin (Tsuneki et al. 2017), partially inhibited the effect of YAP1 in suppressing apoptosis induced by HG in cardiomyocytes (Fig. 6d).

## Discussion

Observational epidemiologic studies have shown that gestational diabetes mellitus is a risk factor for congenital heart anomalies, but the molecular basis of CHD resulting from pregestational diabetes remains obscure. Thus, identification and characterization of novel genes and proteins associated with pregestational diabetes-associated CHD remains an important task. Here, we observed increased MST1 protein levels in the fetal heart tissue of rats exposed to diabetes (Fig. 1a–c). Our in vitro results also revealed increased protein and mRNA levels of MST1 in cardiomyocytes after exposure to HG (Fig. 1c, d). The MST1 over-expression and MST1 siRNA plasmids were employed for further study. Apoptosis assays showed that suppression of endogenous MST1 reduced HG-induced cardiomyocyte apoptosis (Fig. 2b, c), while overexpression of MST1 increased the numbers of apoptotic cardiomyocytes (Fig. 2e, f). These results supported the requirement of MST1 for HG-mediated cardiomyocyte apoptosis Zhang et al. (2016) reported that MST1 regulates apoptosis in diabetic cardiomyopathy in adults. Cardiac development is a complex process involving the differential expression of many genes, and there are differences in the structure and function between the fetal and adult heart. Thus, we are interested in examining the expression of MST1 and its downregulators in the embryonic heart in response to HG.

YAP1 is a core factor of the Hippo pathway (Vita et al. 2018). Although our results demonstrated that decreased YAP1 protein levels in cardiomyocytes in response to HG in vivo and in vitro (Fig. 3a–c), there was no obvious change in the YAP1 mRNA level by qRT-PCR (Fig. 3d). These results suggested that the decrease in YAP1 protein in the HG environment is regulated at the level of translation, and not transcription. Our results showed that MST1 decreased the protein level of YAP1 through changing its phosphorylation level at Ser127 and Ser397 (Fig. 5a). Phosphorylation of Ser127 reportedly leads to the retention of YAP1 in the cytoplasm, where it undergoes further phosphorylation and ubiquitination-dependent degradation. Phosphorylation of residue Ser397 also leads to ubiquitination of YAP1, but via the Skp1–Cullin1–F-box protein β-transducin repeats-containing proteins pathway (Britschgi et al. 2017; Jang et al. 2017; Zhao et al. 2010; Varelas 2014). Previous studies reported that phosphorylation of YAP1 at Ser127 and Ser397 was directly mediated by LATS1/2 (Jang et al. 2017; Zhao et al. 2010; Varelas 2014). There are also many reports of MST1 mediating Lats1/2 phosphorylation (Meng et al. 2015; Hong et al. 2016), which were confirmed by the results of this study (Fig. 5b). Taken together, our results suggested that MST1 indirectly mediates phosphorylation of YAP1 at Ser127 and Ser397 through Lats1/2 in response to HG, and phosphorylation of YAP1 at Ser127 and Ser397 sites led to its ubiquitination and low protein level in response to HG.

YAP1, as a transcriptional modulator, mediates cell proliferation and apoptosis through many target genes, such as CyclinD1 (Wong et al. 2016) and Survivin (Rosenbluh et al. 2012), in response to stressors in different cell types. Our results showed that the protein level of Survivin was decreased under exposure to HG in cardiomyocytes, but there was little change in CyclinD1 protein. Moreover, the decrease in Survivin was inhibited by overexpression of YAP1 (Fig. 6c). These results suggested that Survivin is the target gene through which YAP1 mediates cardiomyocyte apoptosis in response to HG. Furthermore, treatment with the Survivin inhibitor YM155, partially inhibited the YAP1-mediated suppression of apoptosis induced by HG in cardiomyocytes (Fig. 6d). Based on the results of our experiments, Survivin inhibitors such as YM155 promotes cardiomyocyte apoptosis, Survivin inhibitors are harm for pregnant woman, and Survivin up-reguation or activation as a potential therapy to protect against apoptosis induced by MST1 in the setting of Gestational diabetes mellitus and HG. We plan to conduct more detailed studies in this area in the future.

## Conclusion

In the present study, we analyzed the role and mechanism of MST1, Lats1/2,YAP1 and Survivin in maternal diabetes-induced CHD and HG-induced cardiomyocyte apoptosis. As shown in Fig. 6e, our current results revealed that increased MST1 protein levels occurred with HG-induced cardiomyocyte apoptosis in the heart tissues of the offspring of diabetic rats in vitro and in vivo respectively. MST1 played a key role in mediating HG-induced apoptosis of cardiomyocytes. Downregulation of YAP1 was associated with MST1-mediated cardiomyocyte apoptosis in response to HG. MST1 downregulated the protein level of YAP1 through mediation of YAP1 phosphorylation on Ser127 and Ser397 in cardiomyocytes, and this process required Lats1/2 participation. MST1 overexpression increased the phosphorylation level of Lats1/2 in vitro, while Lats1/2 phosphorylation level was increased in the heart tissues of diabetic offspring. Furthermore, we found that YAP1 mediated the expression of Survivin during HG-induced apoptosis, and Survivin-inhibitor YM155 partially inhibited the role of YAP1 in suppressing HG-induced apoptosis in cardiomyocytes. Collectively, this study revealed the expression and roles of MST1, YAP1, and Lats1/2, and their downstream gene Survivin, in modulating cardiomyocyte apoptosis and maternal diabetes-induced abnormalities.

## Supplementary Information

**Additional file 1:** Increase protein level of MST1 in the cardiomyocytes of diabetic offspring in vivo and vitro, respectively.

**Additional file 2:** Quantitative analysis of MST1 protein expression in cardiomyocytes.

**Additional file 3:** Decrease protein level of YAP1 in the cardiomyocytes of diabetic offspring in vivo and vitro, respectively.

**Additional file 4:** Quantitative analysis of YAP1 protein expression in cardiomyocytes.

**Additional file 5:** Quantitative analysis of the YAP1 and LATS1/2 phosphorylation level in cardiomyocytes, respectively.

**Additional file 6:** Quantitative analysis of Survivin and Cyclin D protein expression in cardiomyocytes, respectively.

## Data Availability

All data generated or analyzed during this study are included in this published article.

## Abbreviations

CHD: Congenital heart disease
MST1: Mammalian sterile 20-like kinase 1
LATS1/2: Large tumor suppressor kinases 1 and 2
YAP1: Yes-associated protein 1
TAZ: Transcriptional coactivator with PDZ-binding motif
AMPK: AMP-activated protein kinase
HG: High glucose
siRNA: Small interfering RNA
qRT-PCR: Quantitative real-time PCR
IHC: Immunohistochemistry
