# Supplementary figures and images for: Elevated MST1 leads to apoptosis via depletion of YAP1 in cardiomyocytes exposed to high glucose
Source: Mol Med. 2021 Feb 10;27:13. doi: 10.1186/s10020-021-00267-6 (PMC7874454; doi:10.1186/s10020-021-00267-6)

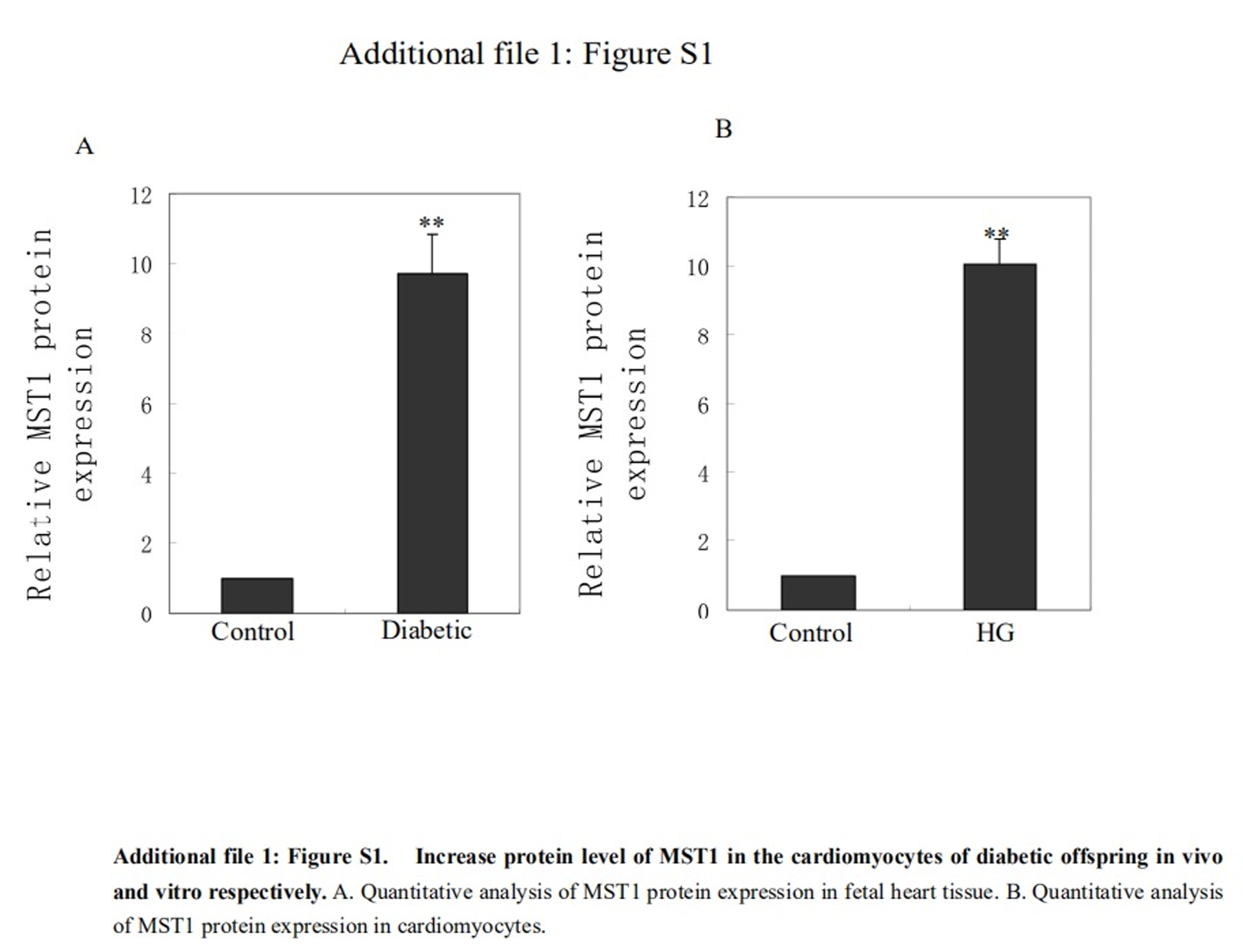

Supplement: Supplementary file 1 — Additional file 1: Increase protein level of MST1 in the cardiomyocytes of diabetic offspring in vivo and vitro, respectively. [file 10020_2021_267_MOESM1_ESM.tif]

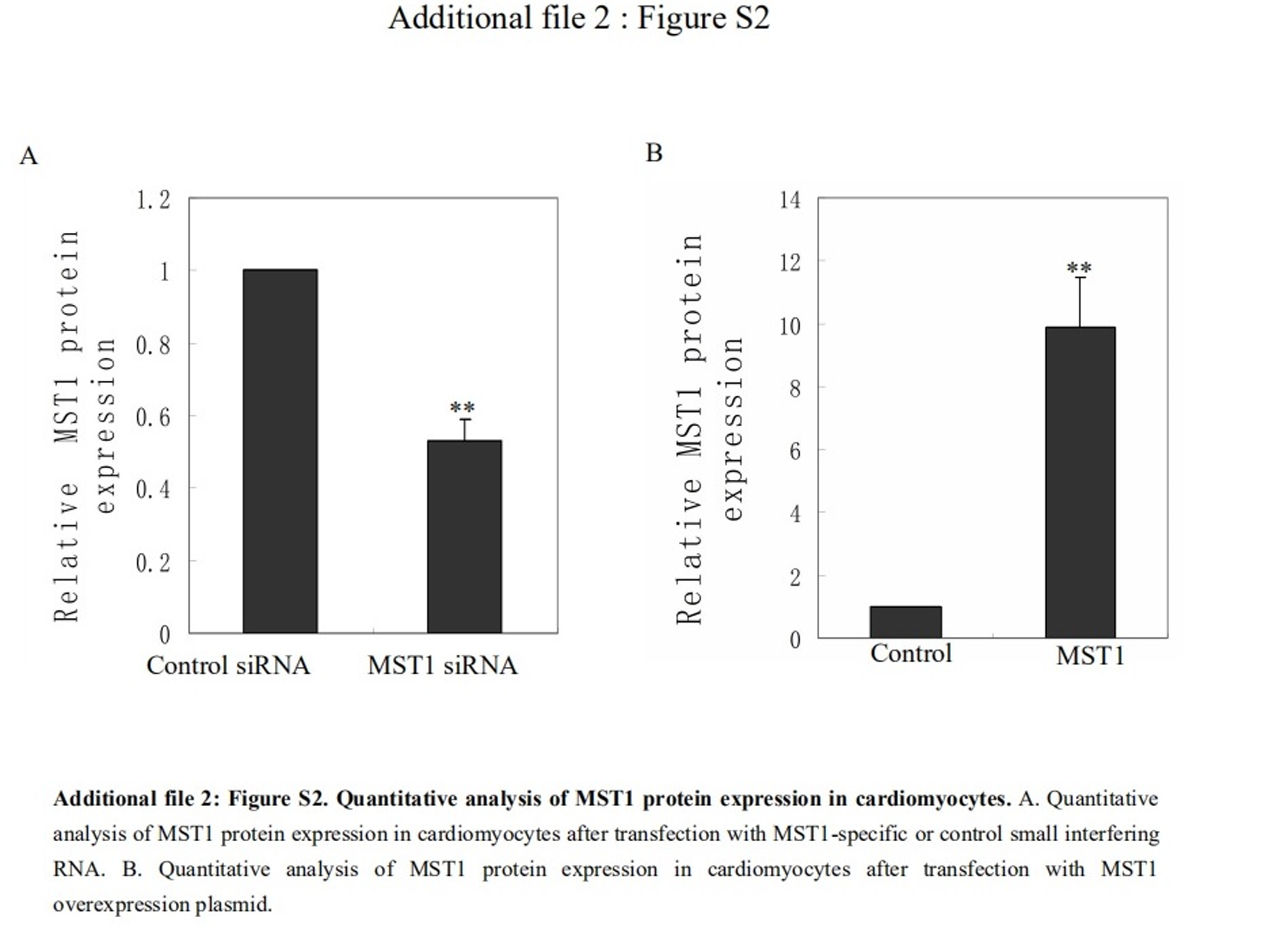

Supplement: Supplementary file 2 — Additional file 2: Quantitative analysis of MST1 protein expression in cardiomyocytes. [file 10020_2021_267_MOESM2_ESM.tif]

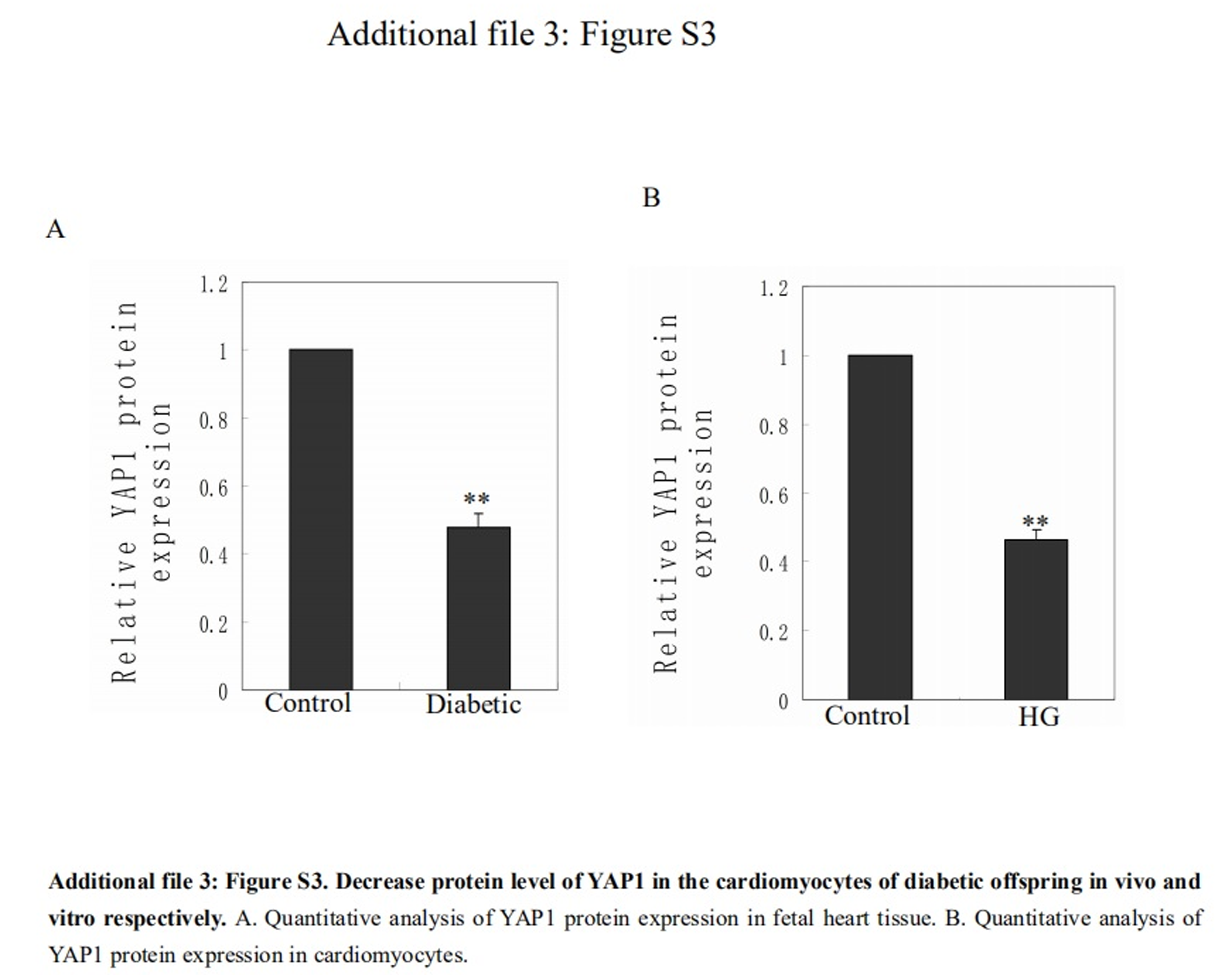

Supplement: Supplementary file 3 — Additional file 3: Decrease protein level of YAP1 in the cardiomyocytes of diabetic offspring in vivo and vitro, respectively. [file 10020_2021_267_MOESM3_ESM.tif]

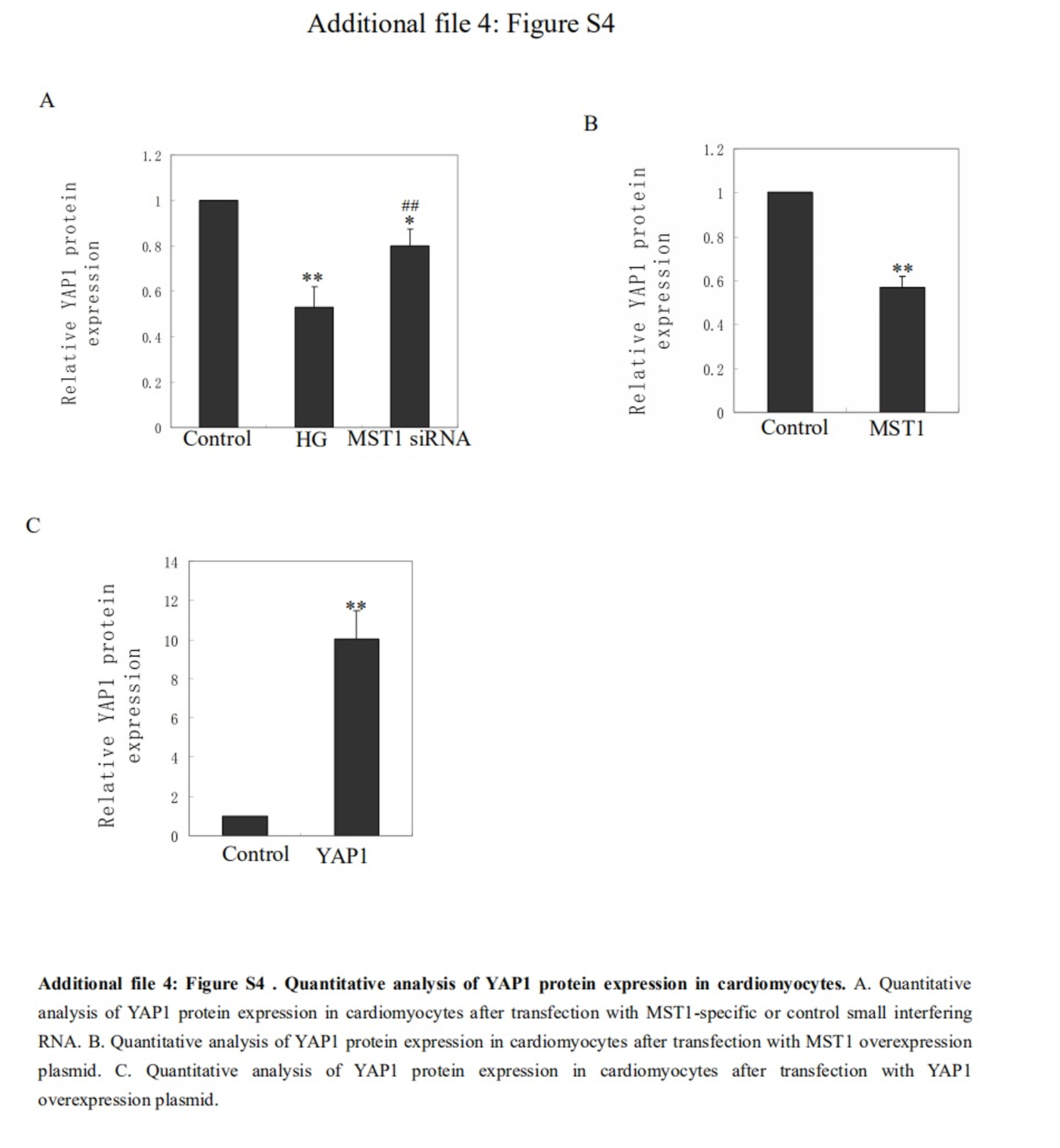

Supplement: Supplementary file 4 — Additional file 4: Quantitative analysis of YAP1 protein expression in cardiomyocytes. [file 10020_2021_267_MOESM4_ESM.tif]

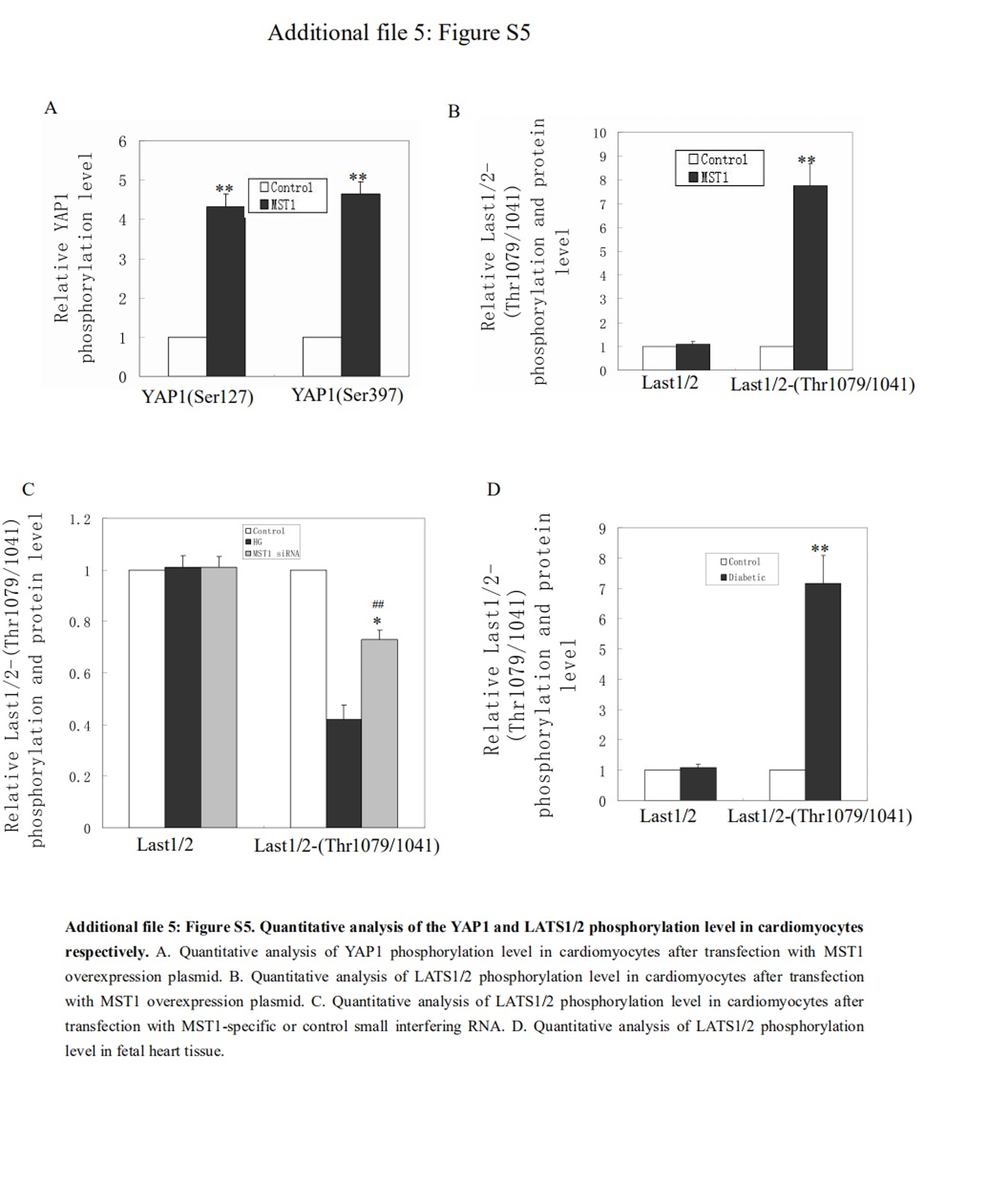

Supplement: Supplementary file 5 — Additional file 5: Quantitative analysis of the YAP1 and LATS1/2 phosphorylation level in cardiomyocytes, respectively. [file 10020_2021_267_MOESM5_ESM.tif]

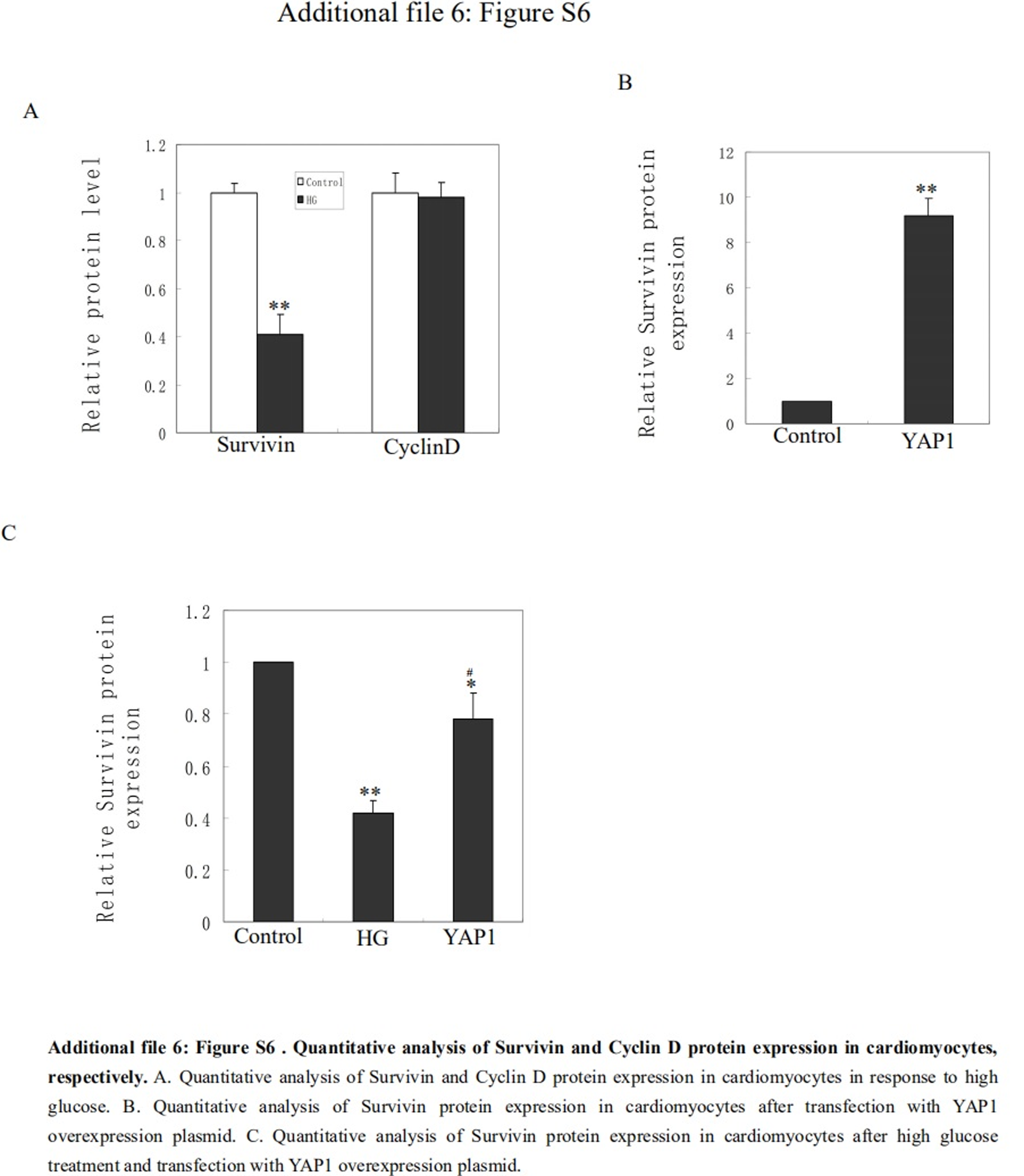

Supplement: Supplementary file 6 — Additional file 6: Quantitative analysis of Survivin and Cyclin D protein expression in cardiomyocytes, respectively. [file 10020_2021_267_MOESM6_ESM.tif]
